# Acute Myeloid Leukaemia With Morphologic and Immunophenotypic Differentiation to Acute Erythroid Leukaemia at the Time of Relapse

**DOI:** 10.1111/ijlh.14386

**Published:** 2024-10-15

**Authors:** Katie Liston, Vitaliy Mykytiv

**Affiliations:** ^1^ Haematology Department Cork University Hospital Ireland

A 58‐year‐old man presented with progressive fatigue and pancytopenia. Blood film analysis revealed a peripheral blast count of 25%. Medical history included untreated localized prostate cancer and a significant smoking history.

Bone marrow aspirate analysis confirmed a morphological diagnosis of acute myeloid leukaemia (AML) with 70% myeloblast infiltration (Figure 1A: 60× objective, Wright‐Giemsa stain). There was single lineage erythroid dysplasia with irregular nuclei and nuclear cytoplasmic asynchrony. Trephine biopsy demonstrated 85% myeloblasts, which stained positively for CD34 and CD117 and negatively for MPO and glycophorin A (Figure 1B: 20× objective, haematoxylin–eosin stain).

**FIGURE 1:**
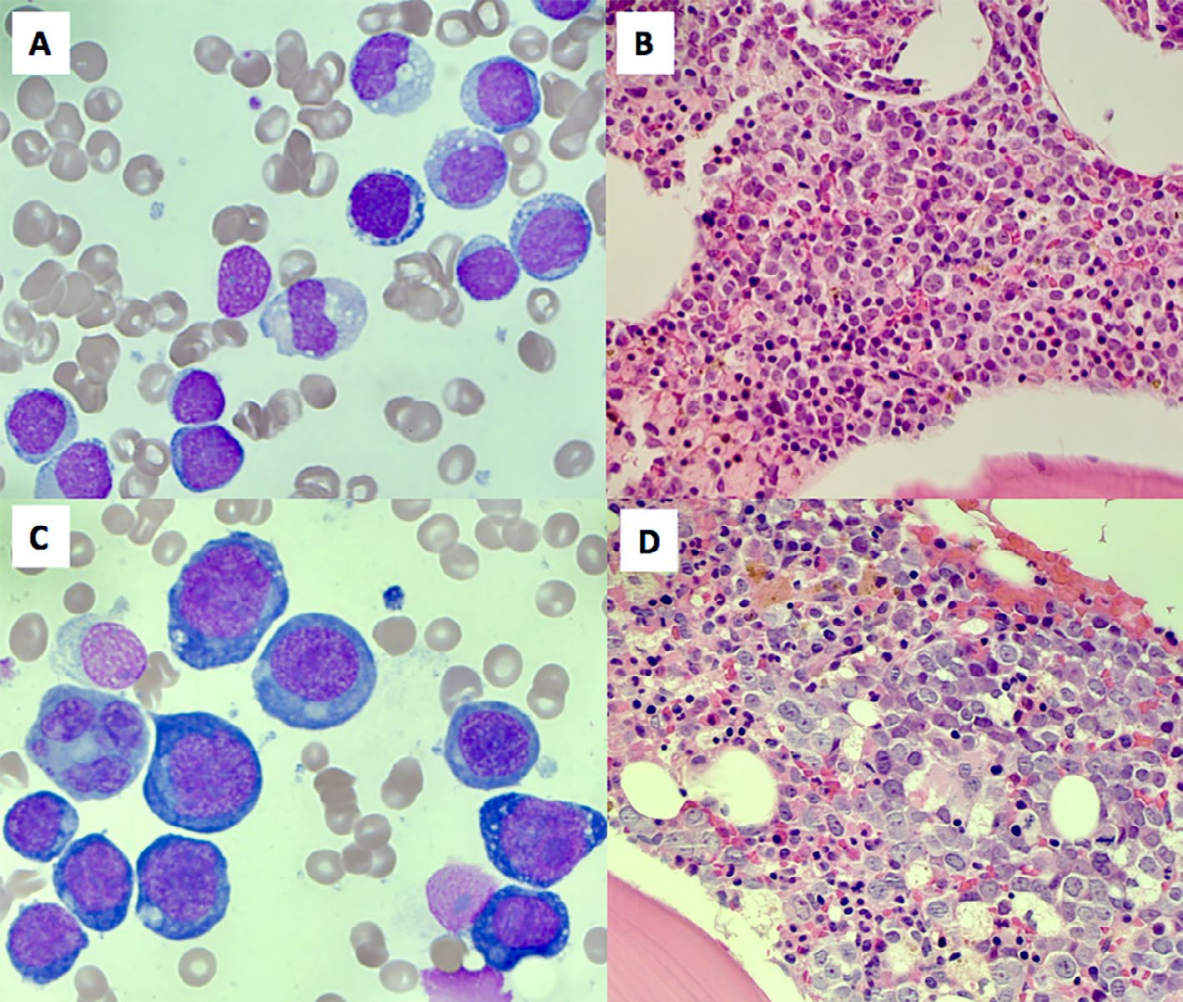
(A) 60× objective, Wright‐Giemsa stain of bone marrow aspirate demonstrating small to moderate size myeloblasts with blue–grey cytoplasm and diffuse chromatin. (B) 20× objective, haematoxylin–eosin stain of bone marrow trephine demonstrating an infiltrate of medium‐sized blast forms with irregular nuclear contours. (C) 60× objective, Wright‐Giemsa stain of bone marrow aspirate demonstrating large immature forms with vacuolated, basophilic cytoplasm and prominent nucleoli consistent with proerythroblasts. (D) 20× objective, haematoxylin–eosin stain of bone marrow trephine demonstrating atypical large blast cells with evenly distributed blastoid chromatin and prominent nucleoli.

Immunophenotyping demonstrated 60% myeloblasts expressing CD34, CD117, CD13, CD33, CD71 and negative for CD36 and CD105. Next generation sequencing (NGS) detected a mutation in the TP53 gene: c.734G>A (p.Gly245Asp) with a variant allele frequency (VAF) of 0.55 and in the NF1 gene: c.4084C>T (p.Arg1362*) with a VAF of 0.23. Cytogenetic analysis revealed a complex karyotype: 42~43,X,−Y,−3,del(5)(q15q33),−13,−17,−18,−21,+mar1,+mar2,inc[cp6]/46,XY[1]. Findings were consistent with AML, myelodysplasia related as per the WHO 2022 classification system and AML with mutated TP53 as per the International Consensus Classification (ICC).

The patient was treated with intensive chemotherapy with fludarabine, cytarabine and idarubicin plus venetoclax. A morphological remission was achieved after the first cycle and a second cycle of therapy was given. Four months after diagnosis, bone marrow analysis revealed relapsed disease. The patient went on to receive decitabine and venetoclax therapy. Unfortunately, there was a limited response and the patient subsequently died 6 months after diagnosis.

At the time of relapse, bone marrow analysis revealed > 80% erythroid predominance with persistent single lineage erythroid dysplasia. There was an infiltration of 65% proerythroblasts. These blasts were morphologically distinct from the myeloblasts seen at diagnosis with evidence of larger forms with vacuolated, basophilic cytoplasm and prominent nucleoli (Figure 1C: 60× objective, Wright‐Giemsa stain).

Trephine biopsy stained negatively for CD34 and MPO (Figure 1D: 20× objective, haematoxylin–eosin stain). Immunophenotyping demonstrated 80% blasts expressing erythroid markers including CD36, CD45 (weak), CD71 (strong), CD105 and Glycophorin A. The blasts did not express CD34. Repeat karyotype analysis showed further progression in karyotypic complexity: 55~60,XY,+1,+2,+del(5)(q13q33),+6,+6,+8,del(13)(q12q21),+14,+14,+15,+16+16,+19,+19,+20,+21,+del(22)(q12),+mar1,+mar2,+mar3[cp6]/46,XY[5]. Repeat NGS demonstrated persistence of mutations identified at diagnosis but at higher VAFs.

According to the 2022 WHO criteria, acute erythroid leukaemia (AEL) (previously classified as pure erythroid leukaemia) is characterised by ≥ 30% proerythroblasts with an erythroid predominance of ≥ 80% in the marrow [1]. De novo AEL is rare, representing < 1% of cases of AML [2]. AEL can progress from other myeloid neoplasms such as myelodysplastic syndrome; however, progression from AML to AEL is extremely rare [3].

In this case, both diagnostic and relapse samples were classified as AML with mutated TP53 as per the ICC. This is in contrast to the WHO 2022 classification whereby the diagnostic sample is classified as AML, myelodysplasia‐related and the relapse sample is classified as AEL. The images depicted demonstrate distinct differences in blast morphology supported by immunophenotypic and immunohistochemical analysis. It is important that clinicians remain aware of differences in that exist between modern classification systems as well as the morphological and immunophenotypic features associated with AEL.

## Author Contributions

Katie Liston is first author on this manuscript. Liston contributed to the data collection, research and reference selection and the writing and editing of this manuscript. Dr. Vitaliy Mykytiv is second author on this manuscript. Mykytiv was the primary care provider for this patient and provided senior oversight in the writing and editing of the manuscript.

## Ethics Statement

The authors have nothing to report.

## Consent

Patient consent was not obtained as patient identifiers were not included.

## Conflicts of Interest

The authors declare no conflicts of interest.

## Data Availability

The data that support the findings of this study are available from the corresponding author upon reasonable request.
